# Liposomal Co-Entrapment of CD40mAb Induces Enhanced IgG Responses against Bacterial Polysaccharide and Protein

**DOI:** 10.1371/journal.pone.0002368

**Published:** 2008-06-04

**Authors:** Caterina Hatzifoti, Andrew Bacon, Helen Marriott, Peter Laing, Andrew W. Heath

**Affiliations:** 1 Adjuvantix Ltd, Sheffield, United Kingdom; 2 Lipoxen Ltd, London, United Kingdom; 3 Unit of Respiratory Medicine, The University of Sheffield, Sheffield, United Kingdom; 4 Unit of Infection and Immunity, The University of Sheffield, Sheffield, United Kingdom; Instituto Oswaldo Cruz and FIOCRUZ, Brazil

## Abstract

**Background:**

Antibody against CD40 is effective in enhancing immune responses to vaccines when chemically conjugated to the vaccine antigen. Unfortunately the requirement for chemical conjugation presents some difficulties in vaccine production and quality control which are compounded when multivalent vaccines are required. We explore here an alternative to chemical conjugation, involving the co-encapsulation of CD40 antibody and antigens in liposomal vehicles.

**Methodology/Principal Findings:**

Anti-mouse CD40 mAb or isotype control mAb were co-entrapped individually in cationic liposomal vehicles with pneumococcal polysaccharides or diphtheria and tetanus toxoids. Retention of CD40 binding activity upon liposomal entrapment was assessed by ELISA and flow cytometry. After subcutaneous immunization of BALB/c female mice, anti-polysaccharide and DT/TT responses were measured by ELISA. Simple co-encapsulation of CD40 antibody allowed for the retention of CD40 binding on the liposome surface, and also produced vaccines with enhanced imunogenicity. Antibody responses against both co-entrapped protein in the form of tetanus toxoid, and *Streptococcus pneumoniae* capsular polysaccharide, were enhanced by co-encapsulation with CD40 antibody. Surprisingly, liposomal encapsulation also appeared to decrease the toxicity of high doses of CD40 antibody as assessed by the degree of splenomegaly induced.

**Conclusions/Significance:**

Liposomal co-encapsulation with CD40 antibody may represent a practical means of producing more immunogenic multivalent vaccines and inducing IgG responses against polysaccharides without the need for conjugation.

## Introduction

In recent years there has been a steady move to better-defined ‘subunit’ vaccines which tend to be safer but less immunogenic than their cellular counterparts. Subunit vaccines require adjuvants in order to be efficacious, but the only adjuvants widely approved for human use, aluminium salts, are not very effective. Safe and potent immunological adjuvants will have applications in a number of areas ranging from prophylactic immunization against infectious diseases through to therapies for allergy, autoimmune diseases and cancer. New adjuvants that are both safe and powerful comprise an enabling technology which will make new vaccines possible, that would otherwise fail due to lack of efficacy.

Ligation of CD40 by CD154 is pivotal to the delivery of T cell help to B cells, leading to immunoglobulin class-switching in both humans and mice [1], [2]. In addition to its importance in T- B interactions, ligation of CD40 is also very important in activation of macrophages and of dendritic cells to express co-stimulatory molecules and thus in the generation of helper T cell priming by these antigen-presenting cells [3]. In recent studies we have shown that ligation of CD40 by antibodies can effectively replace ligation by CD154 expressed on activated T cells. We have shown that large doses of anti-CD40 (500 µg/mouse) are able to induce strong, class-switched antibody responses against T independent (TI) antigens including pneumococcal polysaccharides [4], [5] and to a lesser extent, TD antigens (unpublished) when injected *mixed* with antigen. However such high doses induce unacceptable side effects and would be impractical for use in prophylactic vaccination. We therefore sought a means: i) to reduce the dose of antibody required; and ii) to enhance the adjuvant effect. We found that by joining together a stimulatory CD40-antibody with antigen (either covalently or non-covalently) we can achieve both of these aims together, using 50–500-fold less antibody to generate a very strong antigen-specific immune response [6], [7], [8].

Vaccines increasingly are required to be ‘multivalent’ – i.e. containing antigens from several different strains of a pathogen (as for influenza virus and the polysaccharide vaccines against *S. pneumoniae*), or containing multiple proteins from a single pathogen that are additive or synergistic in the protective immune response they generate (as is the case for a number of vaccines under development – e.g. for *H. pylori*, the new Meningococcal Group B vaccine etc.). There is also an economic and public health case for combining vaccines against different pathogens in a ‘multivalent’ format (e.g. DTP-Hib) in order to increase uptake by the population, and to reduce the total number of injections required to induce protection against the widest possible range of infections and thus reduce the cost of vaccination programmes. Unfortunately the production of antibody-antigen conjugates is a procedure which has to be specially adapted for each antigen, and the production of multivalent conjugate vaccines has proven expensive. We have therefore sought an alternative method that would allow for the close-association of CD40 antibody and antigen, without the need for physical conjugation.

We considered that a possible means of both producing close antigen-CD40mAb association, and producing multivalent vaccines with enhanced immunogenicity might be to incorporate the antigen or antigens, along with the CD40 mAb adjuvant, into liposomes. Liposomes were first proposed as vaccine carriers some time ago [9], and have a well established history in the pharmaceutical industry.

We describe here the production of liposomes containing entrapped CD40mAb along with antigens, either polysaccharide or protein. We show that the CD40mAb containing liposomes are able to bind to CD40, and confer enhanced immunogenicity on the entrapped antigen, while entrapment of the CD40mAb also serves to reduce its toxicity.

## Results

### Liposomal entrapment of polysaccharides and CD40 mAb

High entrapment efficiencies for all antibody materials were seen (average % entrapment 94.3, 99–89.6, (95% CI limits)) and there was no significant variance in entrapment depending on whether the antibody was co-entrapped with PS. The PS entrapment efficiencies were 88% and 60% for PS alone, or entrapped with antibody.

### Function of entrapped CD40 mAbs

**Figure 1:**
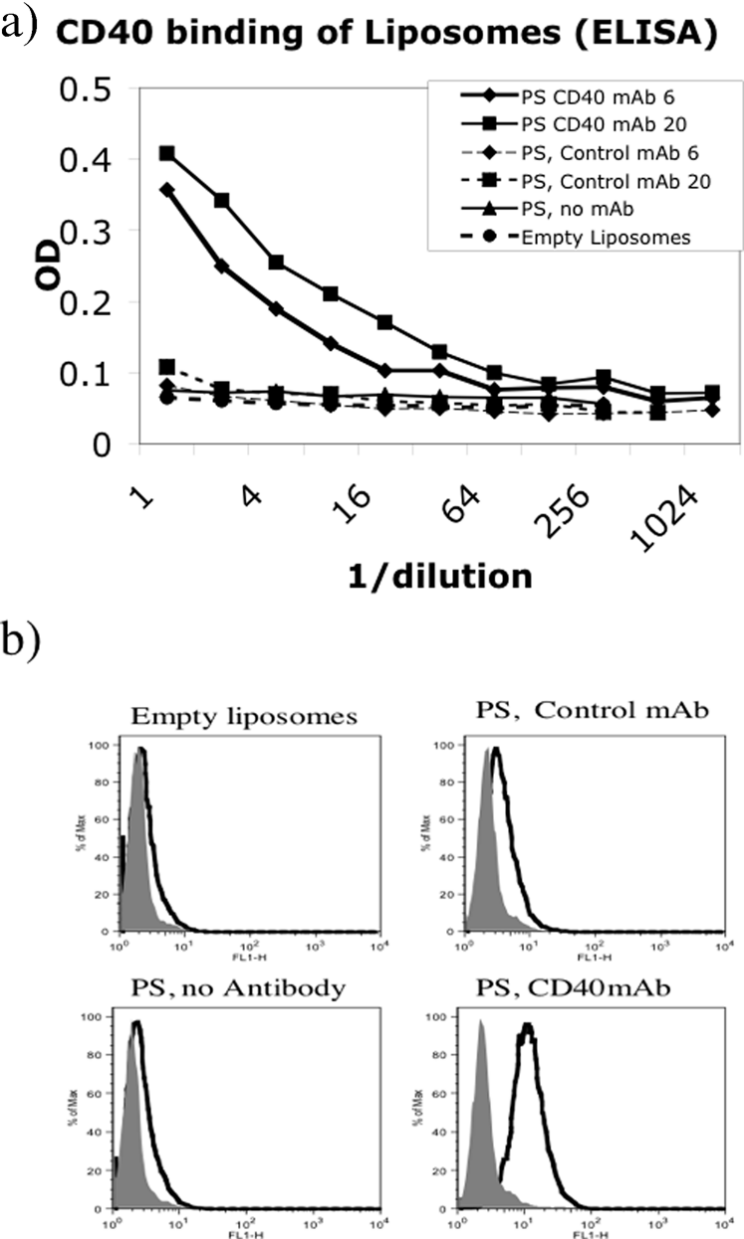
Assessment of liposome CD40 binding activity. Fig 1a) ELISA plates were coated with anti-human IgG, blocked with 3% BSA, and then incubated with recombinant murine CD40-human IgG1. After washing, various liposomal preparations containing entrapped pneumococcal type 3 polysaccharide (PS) and/or CD40 mAb at varying concentrations (6 or 20 µg per 0.5 ml of liposomal preparation) were added to the plate in two-fold dilutions. Liposomal binding to CD40-was detected using HRP labelled goat anti-rat IgG. 1b) To assess liposomal binding to cell surface CD40, CD154 transfected (filled histograms) or CD40 transfected (open histograms) L929 cells were stained with liposomal preparations at a 1/10 dilution in FACS buffer. Binding was detected using FITC labelled anti-rat IgG.

Because CD40 antibody conjugates are thought to bind directly to antigen specific B cells [8], and because it was unknown whether simple liposomal formulation of antibody would allow it to bind CD40, initial experiments were performed to assess the effectiveness of simple liposomal formulations in binding to CD40. Assessment of CD40 binding by the liposomes was performed by Flow cytometric analysis on CD40 transfected or normal L929 cells, and by ELISA assay using plates coated with recombinant murine CD40-Fc, and with detection in both cases by anti-rat antibody conjugates (the monoclonal antibody is rat anti-mouse CD40). The two assays were consistent in that the CD40mAb containing liposomes clearly were able to bind to both recombinant CD40-Fc (Figure 1a), and to cell expressed CD40 (Fig 1b). While the binding found in the ELISA assay may have been attributable, at least in part, to leakage of the liposomal contents in the presence of Tween, the Flow cytometric staining was done in the absence of detergent. The liposomes are stable even at room temperature in the absence of Tween. In a separate experiment less than 1% of entrapped carboxyfluorescein was released over a 20 minute incubation even at room temperature (not shown). These observations, taken together, are consistent with the ability of liposomal formulations to entrap proteins both within the aqueous core and in the lipid membrane of the liposome.

### Responses to protein/CD40mAb liposomes

**Figure 2:**
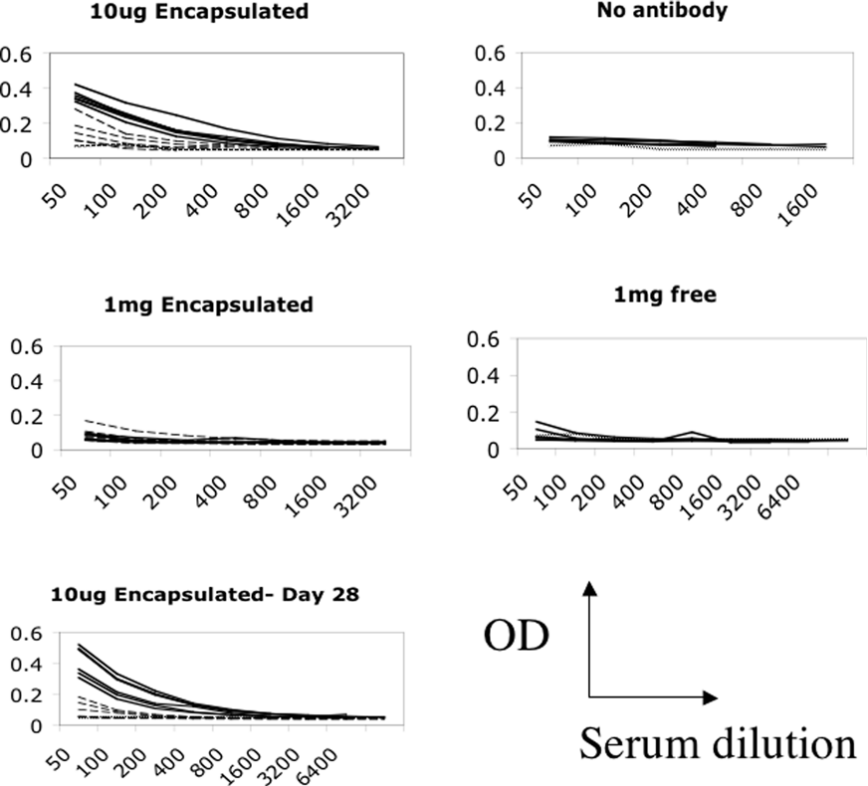
Immune responses against Tetanus toxoid. Immune responses against Tetanus toxoid induced by TT/DT co-encapsulated in liposomes (3 µg DT, 1 µg TT) along with either 10 µg or 1mg of CD40mAb (solid lines) or isotype control mAb (dashed lines). On the right hand side the effect of 1mg of free antibody mixed with 1 µg TT are shown. All plots are ELISA assays on individual sera. All results day 14 post-injection except for the bottom graph, which is day 28.

Tetanus toxoid (TT) and diphtheria toxoid (DT), or the mutant form of diphtheria toxin, CRM197, are currently administered as components of multivalent formulations, such as DTP (along with a pertussis component) or DTP-Hib (with pertussis and *Haemophilus influenzae* conjugate). These vaccines are administered many times in order to achieve and maintain sufficient immune responses (the DT components are given five times in the UK before age 18). Clearly improvement in the immunogenicity of the DT components, which could lead to a reduction in the number of doses required would be highly desirable. Liposomes were formulated and mice immunized with the equivalent of 5 µg of CRM197 (DT) and 1 µg of TT, along with doses of CD40mAb starting at 10 µg encapsulated in liposomes. At day 14, following a single immunization, antibody responses against TT were enhanced in the CD40mAb group as compared with the isotype control liposome group (Fig 2, Table 1, p = 0.0011, Student's t test for differences in geometric mean endpoint titers (GMT) of 800 CD40mAb group versus 114 for the control group immunised with 10 µg control antibody co-encapsulated in liposomes (group 2). None of the groups other than those immunised with 10 µg CD40mAb co-encapsulated with the antigen in liposomes produced responses significantly better than the control group (group 8 in Table 1) immunised with TT alone. (Group 1 vs Group 8 p<0.01. All others vs Group 8: p>0.05 ANOVA with Dunnett's correction for multiple comparisons)

**Table 1:** Antibody titres induced against Tetanus toxoid.

Group	Antigens (µg)	Antibody (µg)	LIPOSOME	GMT (anti-TT)
1	DT (5) TT (1)	CD40 (10)	YES	800
2	DT (5) TT (1)	CONTROL (10)	YES	114
3	DT (5) TT (1)	CD40 (100)	YES	28
4	DT (5) TT (1)	CONTROL (100)	YES	28
5	DT (5) TT (1)	CD40 (1000)	YES	39
6	DT (5) TT (1)	CONTROL (1000)	YES	39
7	DT (5) TT (1)	CD40 (1000)	NO	35
8	TT (1)	NONE	NO	63

This significant enhancement in responses to TT remained at day 28 (fig 2, group 1 GMT 4525, Group 2 GMT 141. p<0.0002) and 45 post-immunization (not shown). Responses to the less immunogenic DT component of the vaccine remained poor, with no response above background detected in any of the groups.

### Responses to polysaccharide/CD40mAb liposomes

**Figure 3:**
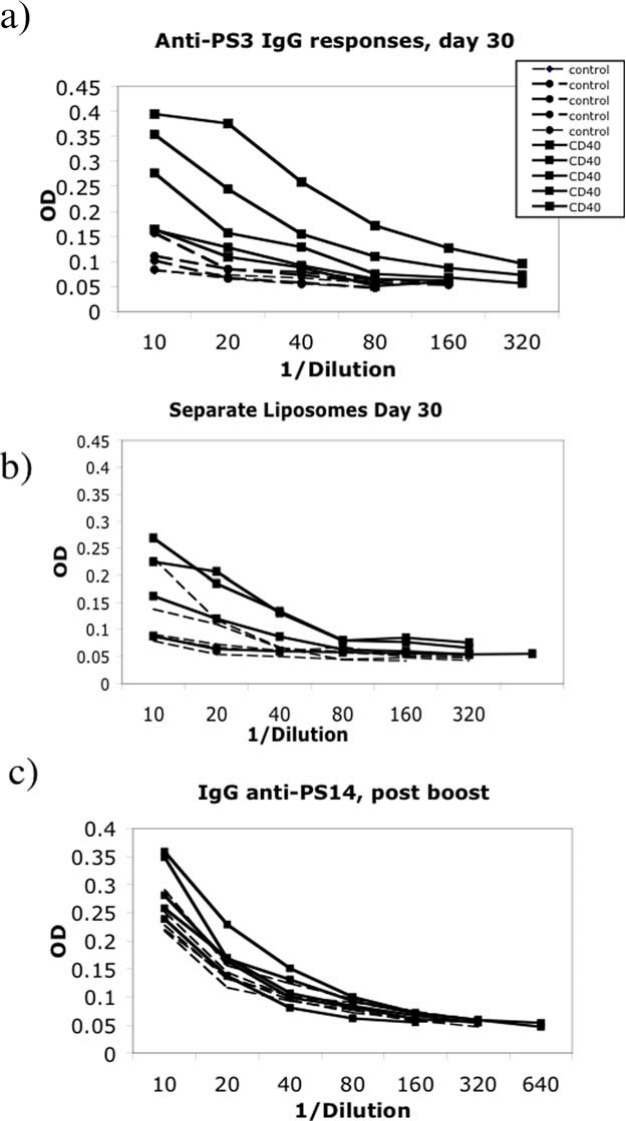
Immune responses against pneumococcal capsular polysaccharides. a) IgG responses at day 30 to PS3 in mice immunized once with liposomes incorporating PS3 (10 µg), PS14 (10 µg) and CD40mAb (10 µg) (solid lines), or PS14, PS3 and control mAb (all 10 µg) (broken lines). CD40mAb/liposome immunised group produced significantly higher titres against PS3 than the control mAb liposome group (IgG GMTs 105 and 11 respectively) p = 0.007, Student's t test. b) IgG responses at day 30 to PS3 in mice immunized once with liposomes incorporating PS3 (10 µg), PS14 (10 µg) and either CD40mAb (10 µg) (solid lines), or control mAb (all 10 µg) (broken lines) incorporated into separate liposomes. Geometric mean titers against PS3 were 11.5 for the control mAb group, and 38 for the CD40mAb group. c) IgG responses 14 days post-boost against PS14 in mice immunized as above. Only two of the 5 mice from the CD40mAb group, and none of the mice from the control mAb group, produced a detectable IgG response against PS14. Overall there was no significant enhancement of responses.

There are over 90 different pneumoccocal capsular polysaccharide types. We incorporated type 3 and type 14 (PS3 and PS14) into multilamellar DRV (dehydration-rehydration vesicle) liposomes. These polysaccharides were chosen as type 3 pneumococcus is one of the most virulent strains, and type 14 is one of the most prevalent. Mice were immunized with liposomes containing 10 µg of antibody and of each polysaccharide, and then antibody responses against PS3 and PS14 assayed by ELISA. Antibody (IgG) responses against PS3 30 days after a single immunization with CD40mAb/PS3/PS14 liposomes, were enhanced in comparison with responses generated by control antibody/PS3/PS14 liposomes (Fig 3a, IgG GMTs against PS3 were 105 in the former group, and 11 in the latter, p = 0.007). There was no significant difference in IgM responses against PS3 (not shown), but in vaccination against T independent antigens an IgG response is highly desirable. In addition, obtaining strong responses after a single immunization is a major aim of all vaccination programmes, albeit a usually unattainable aim. There was little detectable response against the PS14 polysaccharide until after a booster dose was given, when two of the five mice given CD40mAb/PS3/PS14 responded slightly more strongly to PS14 than the controls (Fig 3b). Immunization with entrapped antigen and free CD40mAb, or free polysaccharide and entrapped CD40mAb did not induce a response any stronger than that induced by polysaccharide entrapped with control antibody (not shown).

It was possible that the liposomes were simply acting as slow release vehicle for both antibody and antigen, however the immune response seen when polysaccharide and antibody were entrapped in separate liposomes which were mixed prior to immunisation, was not enhanced to the same extent as it was by immunisation with liposomes in which the CD40 mAb and antigen were co-entrapped (Fig 3b) indicating a requirement for co-entrapment for the best enhancement of immune responses, and likely meaning that a simple “depot effect” was not responsible for the adjuvant effect of co-entrapment.

### Assessment of the toxicity of CD40 liposome preparations

**Figure 4:**
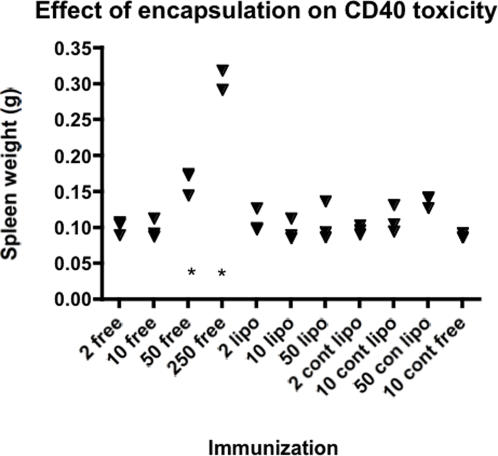
Effect of liposomal entrapment of CD40mAb on toxicity. Increase in spleen size following immunization with liposomally entrapped or free CD40 antibodies. The equivalents of 2, 10 and 50 µg of antibody was injected intraperitoneally into BALB/c mice (3 per group). Spleen weights are shown, the four right hand groups (cont) were injected with isotype control antibody, the rest with CD40 antibody, either free in PBS (free), or encapsulated in liposomes (lipo). Differences in spleen weights were assessed versus 10 µg of free control antibody. Only two groups had significantly enhanced spleen weights (p<0.05, *, ANOVA with Dunnett's correction for multiple comparisons).

We have shown previously that chemical conjugation of CD40mAbs to antigen enhances the adjuvant effect of the antibody, allowing doses of antibody used to be reduced to sub-toxic levels (higher doses induce polyclonal antibody increases and increases in spleen size, which peak at around day 5 post-injection [4] and Newton *et al*, unpublished). As we have shown (above) that the adjuvant effect of CD40 antibody, at the low dose, can be enhanced by liposomal entrapment as well as by conjugation, we were interested in assessing the effects of this entrapment on toxicity. It was theoretically possible that entrapment might increase the toxic effects of the antibody by enhancing receptor cross-linking. In fact, entrapment within liposomes served to decrease the toxicity of the CD40 antibody (Fig 4). While at a 50 µg dose of free CD40mAb there is a significant increase in spleen weight at day 5 (p<0.05), there was no splenomegaly induced by 50 µg of CD40mAb entrapped within liposomes.

## Discussion

Subunit vaccines based upon recombinant proteins or synthetic peptides have very much reduced immunogenicity compared with whole cell vaccines, and require more potent adjuvants than are currently available to enable them to work effectively. Pure polysaccharide antigens are ineffective in major target populations, and polysaccharide conjugate vaccines are expensive to produce, especially in a multivalent form which is often required. We have shown that conjugation of CD40 antibody with protein antigens is an effective means of enhancing immunogenicity [6], [7], [8], and we have also shown that high doses of CD40 antibody can enhance the response to T-independent, polysaccharide antigens [4]. Unfortunately the conjugation of CD40 mAb to antigen is a relatively costly process which becomes more difficult to quality control with increasing numbers of antigens. In this study we aimed to assess a novel method for producing “virtual conjugates” of CD40 mAb with antigen, which would produce enhanced immunogenicity in the absence of physical conjugation, and which would also allow for the production of multivalent vaccines. The method for producing these “virtual conjugates” was to entrap antibody and antigen together in liposomes. A third potential advantage of this approach would be a synergistic or additive effect of the adjuvant effect of liposomes, with the adjuvant effect of CD40mAb.

The presence of CD40 antibody on the liposome surface was assessed by two methods. The first was an ELISA assay designed to assess the binding of liposomes to recombinant murine CD40. The second was a flow cytometric assay to determine whether the liposomes could bind CD40 expressed on transfected L929 cells. Both assays showed that liposomes with encapsulated CD40 mAb had CD40 binding activity on their surface. The degree of binding detected was dependent upon the amount of CD40 mAb entrapped. Non-specific immunoglobulin binding to CD40 or cells was excluded by using isotype control liposomes and normal L929 cells as negative controls.

The oldest form of subunit vaccine is the purified bacterial toxoid. As the two major toxoids in use, diphtheria (DT) and tetanus toxoids (TT), are co-administered, usually along with a third, pertussis component, we incorporated DT and TT into liposomes along with CD40 antibody or its isotype control. Mice were immunized with these liposomal formulations, and antibody responses against the toxoids assessed at various time points. While there was no detectable antibody response against DT in any of the immunized mice, the anti-TT response was enhanced strongly by the co-entrapment of CD40 mAb into the liposomes. CD40 mAb alone had no effect on anti-TT titers when admixed with TT prior to immunization, at either 10, 100 or 1000 µg of antibody. In contrast, just 10 µg CD40 antibody delivered co-entrapped with TT was sufficient to boost the anti-TT response measured a month after vaccination by around 30-fold. A milligram of CD40 antibody entrapped had little effect on immune responses. A similar drop off of adjuvant effects with high doses was seen when using pure CD40 antibody and measuring anti-rat IgG responses (the Fc part of the antibody being effectively linked to the CD40 binding parts) [6]. Of course we did not compare tetanus toxoid immunisation in CD40mAb containing liposomes with the immunisation method used in humans, in which DT and TT are combined with whole or acellular pertussis vaccine for some of the immunisations and with inactivated polio vaccine and Hib conjugate in some countries for some doses. We cannot therefore assume delivery via this method would be superior, just that the inclusion of CD40mAb in liposomes alongside TT imparts an increased adjuvant effect on the liposomal formulation, or in other words, liposomal co-entrapment imparts an adjuvant effect on CD40mAb which is not present when it is simply mixed with the antigen.

Bacterial capsular polysaccharides are the major vaccine candidates for use against encapsulated bacteria, but as they are T independent antigens, conjugation to a protein carrier is normally required to generate a class-switched, IgG response, and to generate a response in infants [10], [11]. Polysaccharide-protein conjugate vaccines against *H.influenzae*, Meningococcus group C, and a 7-valent pneumococcal vaccine are currently on the market. Unfortunately however, there are over 90 capsular polysaccharide serotypes of *S. pneumoniae*, and to produce a conjugate vaccine inducing immunity against all of these would be a monumental task. In fact production difficulties have even prevented the mimicking of the older, 23-valent polysaccharide vaccine by conjugates able to work in infants, and to our knowledge the most multivalent conjugate vaccine assessed to date contains only nine polysaccharides [12]. We had shown previously that high doses of CD40 mAb could induce T dependent-like IgG responses against pneumococcal polysaccharides [4] and also that *conjugates* of CD40 mAbs with TD antigens were effective adjuvants [6], [8], [7]. We were interested therefore in determining whether CD40 mAbs could effectively boost the antibody response against capsular polysaccharides in the absence of conjugation, but when both antibody and polysaccharide were encapsulated in liposomes. Pneumoccal PS 3 and 14 were encapsulated in liposomes along with CD40 mAb or an isotype control, and mice were immunized. Surprisingly, there was an enhanced IgG response induced against PS3 by the CD40 liposomes, and while such a response was not seen against the PS14 component, there was also evidence of some enhancement of this response after boosting. Although there was evidence of an adjuvant effect of CD40mAb, this was not as strong when PS3 was incorporated into separate liposomes from the antibody, indicating that a simple depot effect of liposomal entrapment was not responsible for the bulk of the adjuvant effect seen when antigen and CD40mAb were co-entrapped.

It is uncertain why the responses to PS14 and to DT were lower than the responses to PS3 and TT which were the co-encapsulated “partners”. It is likely that the inherently lower immunogenicity of DT as compared with TT may have played some part, but we also cannot discount the effects of varying entrapment efficiencies, especially with the polysaccharides. PS3 and PS14 are quite different chemically, PS14 is a neutral polysaccharide [13] while PS3 is polar [14]. While we know that, in individual entrapment experiments the efficiency of entrapment of both polysaccharides was high, we cannot exclude the possibility that when dual entrapment was attempted, the entrapment of PS3 was much more efficient than that of PS14. The assays used could not discriminate between the two polysaccharides and indeed this would not be a simple matter to resolve.

Large doses of CD40 antibody, given admixed with polysaccharide or other antigens, are able to enhance both anti-polysaccharide antibody responses [4], and cell mediated immune responses against tumors [15]. These large doses however come with significant toxic effects shortly after the time of delivery [4] and are also linked with a potential suppression of memory responses against tumor antigens which has been attributed to IFN-γ release after administration of CD40mAb [16]. We were concerned that in using liposomal entrapment to enhance what is effectively a non-existing adjuvant effect of this low, apparently non-toxic dose of CD40, we might have concomitantly increased the toxic effect. Somewhat surprisingly the effect of liposomal entrapment was the opposite, in that an otherwise toxic, splenomegaly inducing dose of 50 µg CD40mAb was rendered non toxic (at least in terms of spleen enlargement) by liposomal entrapment (Fig 4).

The most likely explanation for the effectiveness of the CD40/liposome combination relates simply to the fact that entrapment will serve to enhance delivery of the CD40 signal to the same cells presenting the vaccine antigen, in much the same way as we assume the chemical conjugates to be working [6], [17]. The adjuvant effect of the CD40 antibody appears weaker than it is following chemical conjugation to a range of other antigens, perhaps because of the weaker association with antigen and because less of the antibody is available for CD40 binding. However there is also an adjuvant effect of liposomal entrapment itself which adds to the CD40 effect

In summary, co-encapsulation, or even separate encapsulation of CD40mAb in liposomes is a potential means of enhancing the immunogenicity of multivalent and polysaccharide vaccines without the need for conjugation.

## Materials and Methods

### Antibody

The anti-mouse CD40 antibody 10C8 (rat IgG1) has been described previously [18]. The hybridoma producing the isotype control (20C2, anti-human IL12 [19]) was purchased from ATCC. Hybridomas were grown in bioreactors and antibody purified from the supernatant by protein G affinity by Sheffield Antibody Resource Centre, UK.

### Production of liposomes

The lipidic materials egg phospatidyl choline (PC) (99% purity) and DOPE were obtained from Avanti polar lipids, whilst DOTAP was obtained from Merck chemicals Ltd. Pneumococcal polysaccharides (PS) were obtained from ATCC, and diphtheria (CRM197) and tetanus toxoids were kind donations from Dr Umesh Shaligram, State serum Institute of India. The liposomal formulations were cationic in nature, and consisted of PC, dioleoylphosphatidylethanolamin (DOPE), 1,2-dioleoyloxy-3-trimethylammoniumpropane (DOTAP) (4∶2∶1 molar ratio) prepared by the addition of materials to small unilemallar vesicles (SUVs) and processed by the DRV method [20]. The resultant liposomes were MLV in nature with an average size of 448 nm (510–385 nm, 95% CI limits) and appeared to show no significant variance in size whether entrapping protein, polysaccharide or both. The assessment of the entrapment efficiency of the immunization materials involved many different methods. The entrapment efficiency of the proteins was assessed by measurement of the proportion of a radio-labelled (tracer) material in the liposomal pellet and supernatant following ultracentrifugation of the final formulation in a (wash) buffer. With respect to the pneumococcal polysaccharides (type 3 and type 14) the phenol-sulphuric acid assay for total sugar was used, however lipidic interference in this system involved the use of multiple lipid extraction stages to yield reliable results.

Dose levels for all formulations were normalized depending upon entrapment efficiencies.

### Immunizations

BALB/c female mice, aged 8–10 weeks (Harlan, UK) were immunized sub-cutaneously with liposomal preparations as described for each experiment. All experiments were performed according to UK Home Office Regulations for animal care and use.

### ELISA assays

#### CD40 binding of liposomes

ELISA plates were coated overnight with goat anti-human IgG (BD Pharmingen) at 10 µg/ml in PBS. They were then blocked with 3% BSA, and incubated at room temperature with recombinant murine CD40-human IgG1 (1 µg/mL, R&D systems) for 30 minutes, washed with PBS-Tween, and then incubated at RT with doubling dilutions of liposomal preparations in PBS. After 30 minutes, plates were washed again, and incubated with HRP labelled goat anti-rat IgG for 30 minutes. After further washes liposomal binding to CD40 was assessed following the addition of substrate. There was no binding of the anti-human coating reagent to either rat IgG, or anti-rat IgG (or vice-versa).

#### Anti-polysaccharide and DT/TT responses

ELISA plates (Costar, UK) were coated with polysaccharides or proteins at 10 µg/ml in carbonate buffer overnight at 4°C. Plates were blocked for one hour with 1% fish gelatin (Sigma, UK), and after washing with PBS-Tween, serial dilutions of immune sera were made. After a further 1h incubation at RT, and washing, HRP labelled goat anti-mouse immunoglobulin, or anti-mouse IgG were added and the plates incubated for a further hour prior to final wash and the addition of substrate. After 20 minutes incubation plates were read in an Anthos Labtec EIA plate reader.

#### Flow cytometry

Murine L929 fibroblasts stably transfected with either CD40 or CD154 [21] were stained with liposomes as follows. Cells (10^6^ per tube) were pelleted in FACS buffer (PBS, 3% BSA) and were re-suspended in liposomal preparations diluted 1/10 in FACS buffer. After 30 min incubation at 4°C, the cells were washed twice with FACS buffer, and were then incubated with FITC labelled anti-rat IgG (BD Pharmingen) at 10 µg/ml in FACS buffer for 30 minutes at 4°C, washed twice in FACS buffer and then analysed on a FACScalibur™ analyser (Becton Dickinson) running Cellquest™ software
